# Characterization of Physicochemical Properties, Volatile Compounds, and Sensory Attributes of *Tonto*, a Traditional Ugandan Banana‐Based Alcoholic Beverage

**DOI:** 10.1002/fsn3.72071

**Published:** 2026-06-30

**Authors:** Ambrose Atwine, Abel Atukwase, Ivan Muzira Mukisa, Stellah Byakika, Albert Nuwagira, Charles M. B. K. Muyanja, Abubakar Sadik Mustafa

**Affiliations:** ^1^ Department of Food Technology and Nutrition, School of Food Technology, Nutrition, and Bioengineering, College of Agricultural and Environmental Sciences Makerere University Kampala Uganda; ^2^ Department of Plant Sciences, Microbiology, and Biotechnology, School of Biological Sciences, College of Natural Sciences Makerere University Kampala Uganda

**Keywords:** acceptability, aroma, esters, fermentation, methanol, *Tonto*

## Abstract

*Tonto* is a traditional Ugandan alcoholic beverage produced by spontaneous fermentation of banana juice. However, its chemical composition and sensory attributes have not been adequately studied. This study characterized the physicochemical properties, volatile compounds, and sensory attributes of *Tonto* (*n* = 42) produced in Ntungamo and Kalungu districts. During the 60 h fermentation, there was a significant (*p* < 0.05) decline in pH (4.9 ± 0.23–3.58 ± 0.32) and total soluble solids (15.01 ± 1.76–5.15 ± 0.74 °Brix), and a corresponding increase in acidity (0.21% ± 0.07%–0.77% ± 0.64%) and ethanol content (0.0–4.73 ± 1.05 %v/v). Thirty‐eight volatile compounds, mainly esters and alcohols, were identified. Fruity and banana notes dominated in samples from Ntungamo, which also received higher acceptability due to high concentrations of ethyl and acetate esters known for fruity sensory attributes. Principal Component Analysis (PCA) further revealed that fruity esters correlated strongly with overall acceptability. Methanol concentrations varied widely (0–950.5 mg/L), with more than half of the samples, particularly those from Kalungu, exceeding the national safety limit (60 mg/L). These results demonstrate substantial variability in the quality and safety of traditionally produced *Tonto* and highlight the influence of regional differences in fermentation practices and raw material composition. The findings provide a scientific basis for improving and standardizing *Tonto* production through improved hygiene, controlled fermentation, process optimization, and starter culture development to enhance product quality and safety.

## Graphical abstract

Traditional Ugandan *Tonto* is produced through the spontaneous fermentation of banana juice and roasted sorghum. Fermentation decreased pH and total soluble solids while increasing ethanol and titratable acidity. A total of 38 volatile compounds were identified, with esters contributing characteristic fruity and banana‐like aromas. Regional differences influenced flavor profile, sensory acceptability, and methanol content, with Ntungamo samples showing superior sensory quality and lower methanol levels than Kalungu samples. These findings highlight the importance of fermentation control and starter culture development for improved *Tonto* quality and safety.

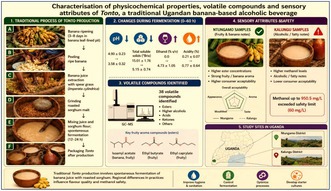

## 1 Introduction

Food fermentation is one of the most ancient methods of food processing that relies on microbial activity to bring about chemical and physical changes in food (Sharma et al. 2020). Fermented fruit beverages hold significant cultural, nutritional, and economic value in many parts of the world where they are used to signify hospitality, social cohesion, and celebration of events like funerals, weddings, rites of passage, and childbirth (Marimo et al. 2019). One such example in Uganda is *Tonto*, a traditional alcoholic beverage made from bananas (*Musa* spp.). Equivalent versions exist in East African countries such as *Urwagwa* in Kenya, Rwanda, and Burundi; *Isongo* in Burundi; and *Mbege* in Tanzania (Marimo et al. 2019; Lyumugabe et al. 2018).

*Tonto* (also known as *Orubisi* or *Mwenge bigere*) is a traditional fermented alcoholic beverage widely produced and consumed in East Africa, particularly in Uganda, Tanzania, and Rwanda (Marimo et al. 2019). It is produced through spontaneous fermentation of juice extracted from beer‐type bananas (*Musa* spp.), including cultivars such as Pisang Awak (locally known as *Kayinja*), *Kisubi*, and *Mbidde* (*Embiire*), which are preferred because of their high fermentable sugar content and juice yield (Rietveld et al. 2013). Beer banana cultivar diversity contributes significantly to the processing characteristics and quality of banana‐based fermented beverages in East Africa. Studies from the Kagera Region of Tanzania have shown that brewing banana cultivars such as *Mbilabile, Ekijoge, Kilomita, Mtwishi, Kalinaba, Kainja, Akanana*, and *Ensharuka* are traditionally preferred for juice extraction and local alcoholic beverage production due to their high juice yield and favorable fermentable sugar characteristics (Msigala et al. 2024). Similarly, *Gisukari* and *Kamaramasenge* cultivars are commonly used in Rwanda because of their suitability for fermentation (Lyumugabe et al. 2018). Blending banana cultivars has also been reported to improve juice yield and beverage quality, highlighting the importance of cultivar selection in determining fermentation performance and final product characteristics (Msigala et al. 2024).

The bunches of bananas are harvested mature and ripened for 3–8 days in a banana leaves lined pit. The ripe bananas are then peeled and mixed with spear grass ( *Imperata cylindrica* ), locally known as *Omuteete* and the juice is extracted by squeezing the mixture with feet in a canoe‐shaped wooden vat (Rietveld et al. 2013; Msigala et al. 2024). Spear grass is a lignocellulosic plant material composed mainly of cellulose, hemicellulose, and lignin, which gives it a fibrous and porous structure suitable for use as a natural filtration and pressing aid during juice extraction. It enhances juice yield and clarity by facilitating drainage, preventing clogging, and retaining coarse particulates. Prior to use, the spear grass is carefully selected to exclude damaged or contaminated material and thoroughly washed with potable water to ensure hygiene and safety. The extracted juice is diluted with water and coarsely ground roasted sorghum is added and left to ferment spontaneously for 12–24 h. After fermentation, the banana beer is filtered again to remove any remaining solids. The final product is a slightly cloudy, mildly alcoholic beverage with a sweet and sour taste. Because of the unstandardized nature of this process, the raw material and final product composition, microorganisms involved, and fermentation conditions can vary across batches, regions, and households.

In traditional *Tonto* production, fermentation occurs spontaneously under ambient conditions, typically in covered wooden vats. Temperatures during fermentation can reach as high as 40°C, reflecting the heat generated by microbial metabolism and the tropical environment Gensi et al. (1996). Wild yeasts and lactic acid bacteria naturally present on the bananas, the wooden vat and added sorghum convert fermentable sugars into ethanol, organic acids and volatile compounds, shaping the beverage's physicochemical and sensory characteristics (Sharma et al. 2020). The fermentation process is influenced by factors such as substrate composition, pH, ambient temperature, and duration, all of which determine the rate of sugar conversion and the development of characteristic flavor, aroma, acidity, and ethanol.

In an effort to standardize banana based fermented alcoholic products in the region, studies have been carried out on the production processes. For instance, improvement of banana juice extraction methods (Gensi et al. 1996), identification of volatile compounds in *Urwagwa*, a traditional banana‐based alcoholic beverage in Rwanda (Lyumugabe et al. 2018). However, there have been no attempts to characterize the flavor compounds in *Tonto* or describe its sensory properties. This study aimed to define the aroma profile and physicochemical properties of *Tonto* at different stages of fermentation in Ntungamo and Kalungu districts in Uganda. The findings from this study will enable commercialisation of *Tonto* while safeguarding the profile of the traditional product.

## 2 Materials and Methods

### 2.1 Study Design and Sample Collection

The study used cross‐sectional, descriptive, and experimental research designs. A cross‐sectional design was used during the collection of traditional fermented *Tonto* samples from Kalungu and Ntungamo districts in the month of March 2023. A descriptive design was used during the characterization of the collected samples by measuring physicochemical parameters, flavor profiles, and sensory attributes. An experimental design was applied in the controlled fermentation experiments and analyses conducted in the Department of Food Technology and Nutrition Laboratories, Makerere University. *Tonto* production is predominantly carried out in central and western Uganda. The two districts were selected for this study due to a high number of *Tonto* processors and being further apart from each other in their geographical location. In Ntungamo, *Tonto* samples were picked from three villages of Rweza (Nt1), Rujumo ward I (Nt2), and Ihunga II (Nt3) located in Rutunguru parish, Kagarama town council, while Kalungu samples were picked from the villages of Serubidde (Ka1), Nantaba (Ka2), and Mutumba (Ka3) located in Kyambala parish, Bukulula sub county. Six *Tonto* producers were selected from the six villages in Ntungamo and Kalungu districts. *Tonto* samples (*n* = 42) were picked from these producers immediately after the initiation of fermentation (*T* = 0) and subsequently at 6, 12, 24, 36, 48, and 60 h. These samples were chilled and transported to the laboratory for further processing and analysis. Spontaneous fermentation trials were also performed in the laboratory following similar methods and conditions as performed by traditional *Tonto* producers in Ntungamo and Kalungu to validate the study results (trial results not shown).

### 2.2 The Traditional *Tonto* Production Process

*Tonto* was produced by fermenting banana juice with coarsely ground roasted sorghum under spontaneous fermentation conditions. The traditional beer banana varieties used in Ntungamo were generally locally referred to as *Embiire* (from the East African Highland Banana beer clone set—EAHB‐AAA). They included *Entundu, Nshakara, Kabula, Kisansa, Kinyika, Rwambarara, Mporogoma, Nyamunuka*, and *Enyeru y'Omwigara*. These are characterized by high levels of fermentable sugars, tannins, bitter‐astringent pulp, sticky brown latex, and high phenolic content, traits that make them particularly suitable for brewing rather than cooking or dessert use.

The beer banana varieties used in Kalungu were generally locally referred to as *Mbidde* and they included *Kayinja* (Pisang awak from ABB‐genome group), *Kisubi* (dessert banana from AB genome group), FHIA 25+ (EAHB‐AAA type banana hybrid), and *Yangambi* KM5 (from the AAA genome group). These were mixed together during juice extraction. These banana varieties were preferred for *Tonto* production in this area because of high juice yield, sweetness (high levels of fermentable sugars), low levels of latex and tannins, and suitability for fermentation.

The bananas were first ripened in a pit lined with banana leaves for 3–8 days, then peeled before being squeezed or pressed in a wooden vat to extract juice using spear grass to facilitate juice extraction. The extracted banana juice was filtered, diluted with water, and mixed with roasted and coarsely ground sorghum. The mixture was then left to ferment naturally at ambient temperature for 60 h. During fermentation, microorganisms, mainly yeasts and lactic acid bacteria, converted sugars into ethanol, organic acids, and flavor compounds, resulting in a mildly alcoholic beverage with characteristic sour–sweet flavor and aroma. During fermentation, samples were picked at 0, 12, 24, 36, 48, and 60 h and analyzed for pH, titratable acidity, total soluble solids, and ethanol content. Volatile flavor compounds and sensory analyses were done on the 24 h *Tonto*, which represents the traditional harvesting and consumption stage of *Tonto*.

### 2.3 Physicochemical Analysis

#### 2.3.1 Total Soluble Solids

Total soluble solids were measured using a handheld Refractometer (ATAGO brand, Japan). Before analysis, the refractometer was thoroughly cleaned and calibrated with distilled water. A drop of each *Tonto* sample was put on the prism surface, covered with the glass plate, and the instrument directed toward natural light. The °Brix value was then read from the vertical scale through the eyepiece and recorded as the total soluble solids (TSS) content of the sample.

#### 2.3.2 pH and Titratable Acidity

*Tonto* pH was determined using a pH meter (INE PHS‐3E model, MRC, Israel) calibrated with standard buffer solutions (pH = 4.0, pH = 7.0 and pH = 10.0). Titratable acidity was determined using the method described in ISO 750 (1998). A volume of 10 mL of the *Tonto* was titrated against standardized 0.1 N NaOH using phenolphthalein indicator. Titratable acidity was then calculated as % lactic acid using Equation (1). An equivalent weight of lactic acid of 90.08 g was used.

(1) $$\%\text{Lactic acid}=\frac{\mathrm{Net}\;\text{titre}\times N\;\text{NaOH}\times 90.08}{\text{Weight of sample}\times 1000}\times 100$$

#### 2.3.3 Ethanol Content

Ethanol content was determined using an Anton Paar Alcolyser Plus Beer Analyzing System (Anton Paar, GmbH, DMA 4500 M, Germany). Calibration and sample analysis followed the manufacturer's instructions. Briefly, samples were centrifuged at 4000 RPM for 5 min, brought to a temperature between 20°C and 22°C, double filtered into clean, dry flasks, and covered with a clock glass to minimize evaporation. Each sample vial was filled to two‐thirds of its volume and covered with the vial cap. Calibration standards (distilled water, 10% v/v ethanol, pH 7 and 14 buffer solutions) were prepared and analyzed using the same procedure. Sample identification was entered using the device's interface, and samples were loaded into the autosampler in the same order as entered. The analysis was initiated using the system's auto‐run function. Cleaning and rinsing vials were included at the end of each run. Ethanol concentration was recorded directly from the system display and expressed as % (v/v).

### 2.4 Volatile Compound Analysis

Volatile compounds in traditionally fermented *Tonto* were identified and quantified using the method described by Lyumugabe et al. (2018). The volatile compounds were extracted using headspace solid‐phase micro extraction method. Each *Tonto* sample (10 mL) was pipetted into 20‐mL round‐bottomed, amber glass headspace vials containing 2.5 g of NaCl and 5 μL of 3‐octanol (internal standard; 100 mg/L in absolute ethanol). The vials were equilibrated at 30°C for 10 min under agitation at 500 rpm. A 50/30 μm divinylbenzene/carboxen/polydimethylsiloxane (DVB/CAR/PDMS) fiber (Supelo, Bellefonte, PA, USA) was inserted and exposed to the headspace of the vial for 30 min with agitation at 250 rpm. Following extraction, the volatile compounds were isolated and identified using gas chromatography–mass spectrometry (GC/MS 7890B, Agilent, USA), coupled with a 5975C inert XL EI/CI mass selective detector (Agilent, USA), equipped with a Thermal Desorption Unit (TDU), a CIS 4 PTV inlet, and an MPS 2 auto sampler with headspace and DHS options (Gerstel, Germany). Here the analytes were first automatically adsorbed in the GC–MS system injection port operated in splitless mode at 250°C for 5 min. The temperature program started at 30°C for 2 min, increasing to 70°C at a rate of 10°C/min and held for 1 min, then increased to 220°C at 4°C/min and held for 2 min and finally to 280°C at 20°C/min with a 6‐min hold. Helium was used as the carrier gas at a constant flow rate of 1.0 mL/min. Mass spectra were acquired in electron impact (EI) mode (mass range: 30–500 m/z) with the ion source temperature set at 230°C. Separation was achieved using an HP‐5MS column (30 m × 0.25 mm ID, 0.25 μm film thickness). Compounds were identified by comparing their retention times and/or spectra with: (i) the standards analyzed under identical conditions, (ii) mass spectra in NIST and Pal1600k.L library databases and (iii) retention indices in literature. All volatile compounds were quantified by the internal standard method. Using threshold levels obtained in literature, odor activity values (OAVs) were calculated to evaluate the contribution of each volatile aroma compound in *Tonto* using Equation (2).

(2) $$\mathrm{OAV}=\frac{\text{Total concentration}\;\left(\mathrm{mg}/L\right)\;\text{of each compound}}{\text{Odour threshold}\;\left(\mathrm{mg}/L\right)}$$

### 2.5 Sensory Evaluation

#### 2.5.1 Acceptability Test

*Tonto* after 24 h fermentation was assessed for acceptability by semi‐trained assessors (*n* = 30), both males (*n* = 20) and females (*n* = 10) from the School of Food Technology, Nutrition and Bioengineering Makerere University, with food science background and experience in *Tonto* consumption. Sensory attributes evaluated were appearance, aroma, flavor, taste, mouthfeel, after taste, and overall acceptability using a 9‐point hedonic scale (1 = Dislike extremely, 2 = Dislike very much, 3 = Dislike moderately, 4 = Dislike slightly, 5 = Neither like nor dislike, 6 = Like slightly, 7 = Like moderately, 8 = Like very much, 9 = Like extremely) as described by Heymann and Lawless (2013). Each *Tonto* sample (50 mL) was coded and provided in duplicate (*n* = 2) and in a randomized order to each panelist in separate booths in the sensory analysis laboratory, Department of Food Technology and Nutrition Makerere University. Panelists cleansed their palate in between using drinking water (mineral water) provided.

#### 2.5.2 Quantitative Descriptive Sensory Analysis (QDA)

Quantitative descriptive sensory analysis was conducted by a trained panel of 10 assessors (5 males and 5 females) at the Department of Food Technology and Nutrition, Makerere University. Panel selection and training followed ISO 13299 guidelines (ISO 13299 2016) and the protocol described by Lawless (2013). Panelists participated in five training sessions covering consensus terminology, aroma recognition, and scale calibration. Firstly, a preliminary assessment using a traditionally fermented *Tonto* sample was conducted to establish relevant sensory descriptors, following ISO 8586‐2 (ISO 8586 2023). Secondly, the panelists discussed, refined, and agreed upon the final set of descriptors (for aroma modalities such as fruity, floral, banana, pineapple, vegetable, and fatty; for taste modalities: sweet, spicy, and alcoholic). The panelists also agreed on a 9‐point quantitative line scale from 1 (lowest intensity) to 9 (greatest intensity). In the final step, they evaluated different *Tonto* samples obtained from Ntungamo and Kalungu at 24 h of fermentation. Each sample (20 mL) was poured into a wine glass and presented at (20°C ± 1°C) in random order in separate booths in the sensory analysis laboratory. Each sample was tested in duplicate (*n* = 2) with an interval of 1–2 min between the samples, and panelists cleansed their palate in‐between using drinking water provided.

### 2.6 Statistical Analysis

Experiments were conducted in duplicate (*n* = 2) and the data presented as mean ± standard deviation. The data generated from characterization of major flavor compounds, sensory analysis, and physicochemical properties of *Tonto* from the six sampling points in Ntungamo and Kalungu districts was organized and analyzed using descriptive statistics. Principal component analysis was done to determine the correlation between the *Tonto* samples, flavor compounds, physicochemical characteristics, sensory characteristics, and detected sensory aroma descriptors. The dendrogram of hierarchical cluster analysis (HCA) and heat map visualizations were applied to visualize the relationship between odor activity values (OAVs) and sensory aroma descriptors. Analysis of variance (ANOVA) and Tukey's test were subjected to the data for determination of variation in the flavor compounds and physicochemical properties at 95% confidence level. The XLSTAT software (version 2023 5.2.1413.0, Addinsoft, Paris, France) was used for the data analysis.

## 3 Results and Discussion

### 3.1 Physicochemical Analysis

**FIGURE 1:**
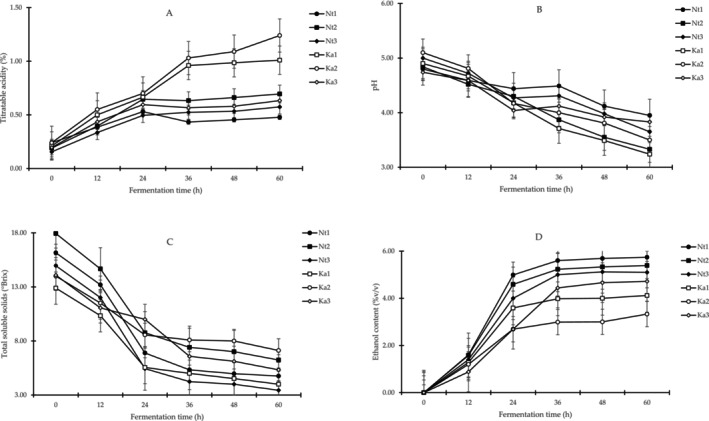
Physicochemical properties of traditionally fermented *Tonto* from Ntungamo and Kalungu districts, at different stages of fermentation. Titratable acidity (A), pH (B), total soluble solids (C), and ethanol content (D). The curves represent the mean of two separate fermentations from the same sampling site while the error bars represent the standard deviation between two replicate determinations. All analyses were done in duplicate.

The changes in pH, titratable acidity, total soluble solids and ethanol content of *Tonto* after 60 h fermentation period are shown in Figure 1A–D. At the start of fermentation, the pH ranged from 4.74 ± 0.18 to 5.10 ± 0.05 (Figure 1B), whereas titratable acidity ranged from 0.16% ± 0.03% to 0.24% ± 0.04% (Figure 1A). As fermentation progressed, there was a consistent drop in pH to values between 3.24 ± 0.01 and 3.95 ± 0.31. This was accompanied by an increase in titratable acidity to values between 0.48% ± 0.09% and 1.24% ± 0.55%. The increase in acidity is likely due to the production of organic acids, predominantly lactic acid and acetic acid, by lactic acid bacteria and other acid‐producing microorganisms during fermentation. These acids accumulate as fermentation progresses reflecting the metabolic activity of fermentative microorganisms, producing organic acids and ethanol, which lower the pH and increase acidity, contributing to acidification, flavor development, microbial stability, and the characteristic sour taste of *Tonto* (Lyumugabe et al. 2012; De Vuyst and Leroy 2020). Total soluble solids varied from 12.89% ± 0.77% to 17.94% ± 0.99% (Figure 1C) at the beginning of fermentation reducing to between 3.44% ± 0.14% and 7.12% ± 0.60% at 60 h of fermentation. The ethanol content increased from 0.0% to a range of 3.33% ± 1.91%–5.74% ± 1.05% during 60 h fermentation (Figure 1D).

The size of the error bars reflects the degree of variability among duplicate fermentations, with larger error bars indicating greater variation and smaller error bars indicating more consistent results. Total soluble solids exhibited the largest error bars during the early stages of fermentation (0–24 h), reflecting variability in the initial sugar content of bananas and differences in sugar utilization rates among fermentation batches. Ethanol content showed the greatest error bars between 24 and 36 h, corresponding to the period of most active alcoholic fermentation, when differences in yeast growth and fermentation efficiency were most pronounced. For pH and titratable acidity, the largest error bars were observed between 36 and 48 h, and 48–60 h respectively, indicating variation in the rate of acid production and buffering capacity among samples. Despite these variations, the overall fermentation trends remained consistent across all samples, demonstrating reproducible fermentation behavior.

Similar trends have been reported in other studies on banana‐based alcoholic fermentations. For instance, Shayo et al. (2000) observed a final pH of 3.73 and titratable acidity between 0.35% and 0.89% in *Urubisi* fermentation. Havyarimana (2021) reported pH values of 4.2–4.58 and titratable acidity ranging from 0.30% to 0.53% in *Tonto* fermentation. Similarly, Lyumugabe et al. (2018) reported pH values between 3.87 and 4.09, and titratable acidity range of 0.541%–0.576% in *Urwagwa*, a traditional banana beer in Rwanda.

Pauline et al. (2017) reported that fermentation progressed with decrease in sugar content with about 40% of the sugar being converted into fermentation products. Similarly, Dhar et al. (2013) reported ethanol content of less than 8% (v/v) whereas Carvalho et al. (2009) indicated a value of 6.39% (v/v) in banana‐based alcoholic beverages. Similarly, *Urwagwa* samples in Ngoma and Rulindo districts of Rwanda had similar total solids content in the range of 4.00 (±0.56) to 4.40 (±1.00) °Brix. However, these samples had high ethanol content ranging from 7.53 (±1.16) to 11.03 (±2.25) % v/v (Lyumugabe et al. 2018). Similar results of total soluble solids and ethanol content were reported in banana juice (10.00 ± 0.00 °Brix; 0.00% v/v), primary fermentation of banana wine (3.53 ± 0.04 °Brix; 5.20% ± 0.14% v/v) and post fermentation of banana wine (4.80 ± 0.14 °Brix; 8.10% ± 0.14% v/v) (Chen et al. 2020).

The physicochemical differences observed between *Tonto* samples from Ntungamo and Kalungu districts may be attributed to variations in banana varieties, microbial populations, environmental conditions, and traditional fermentation practices. Different banana varieties are known to differ in sugar content, dry matter, acidity, mineral composition, and nutrient availability, all of which influence microbial activity and fermentation performance. In the present study, *Tonto* producers from Ntungamo predominantly used locally available brewing banana cultivars with relatively higher sugar content, whereas producers from Kalungu used mixed cultivars depending on seasonal availability. Variations in sorghum composition and preparation methods may also have contributed to differences in fermentation dynamics and final product characteristics.

Additionally, environmental and processing conditions differed between the two districts. Ntungamo district is located in the southwestern highlands of Uganda, characterized by relatively cooler temperatures, while Kalungu district lies within the central region with comparatively warmer conditions. These environmental differences may influence microbial succession and metabolic activities during spontaneous fermentation. Similar regional differences in banana‐based fermentations have previously been associated with variations in raw material composition and microbial ecology (Lyumugabe et al. 2018). *Urwagwa* produced in Rulindo district had a higher ethanol content (11.03%) because it was made using the *Gisukari* variety compared to that produced in Ngoma district (7.3%) made from the *Kamaramasenge* variety.

The physicochemical properties of *Tonto* samples from Ntungamo and Kalungu were checked for compliance with the specifications of US 2143 (Uganda *Tonto* standard) (Uganda National Bureau of Standards 2019). The comparisons are presented in Table 1. It can be noted that samples obtained from Kalungu did not conform to most of the specifications such as ethanol, methanol, and total soluble solids, whereas samples from Ntungamo did not comply with the total soluble solids.

**TABLE 1:** Compliance of 24 h traditional fermented *Tonto* with the Uganda *Tonto* standard (US 2143: 2019).

Characteristics	Nt1	Nt2	Nt3	Ka1	Ka2	Ka3	US 2143: 2019
Ethanol content (%v/v)	5.0^a^ ± 0.88	4.6^a^ ± 0.52	4.0 ± ^ab^0.71	3.6^b^ ± 0.53	2.7^c^ ± 0.71*	2.7^c^ ± 0.62*	4.0–12.5
pH	4.4^a^ ± 0.09	4.3^ab^ ± 0.04	4.3^ab^ ± 0.01	4.2^b^ ± 0.01	4.2^b^ ± 0.06	4.0^c^ ± 0.08	2.5–5.0
Methanol (mg/L) Max.	43.9^c^ ± 0.89	ND	ND	285.4^b^ ± 0.97*	288.2^b^ ± 3.01*	950.5^a^ ± 3.21*	60
Total soluble solids (%)	6.9^b^ ± 1.0*	8.8^a^ ± 0.84*	5.4^c^ ± 1.2	5.6^c^ ± 1.53	8.6^a^ ± 1.24*	10.0^a^ ± 1.33*	3.0–6.0
Total acidity, Lactic acid (%) Max.	0.5^b^ ± 0.4	0.7^a^ ± 0.05	0.5^b^ ± 0.07	0.7^a^ ± 0.73	0.7^a^ ± 0.50	0.6^ab^ ± 0.22	0.28–1.10

* Did not meet the standard specifications. Methanol was analyzed using GC–MS coupled with head space solid phase microextraction as indicated in section 2.4. The data represents the mean of two separate fermentations from the same sampling site and the value after ± represents the standard deviation between two replicate determinations. All analyses were done in duplicate. Compounds with different letters within the same row indicate differences among *Tonto* samples determined by Tukey's HSD test at 95% confidence level.

### 3.2 Volatile Compounds

**FIGURE 2:**
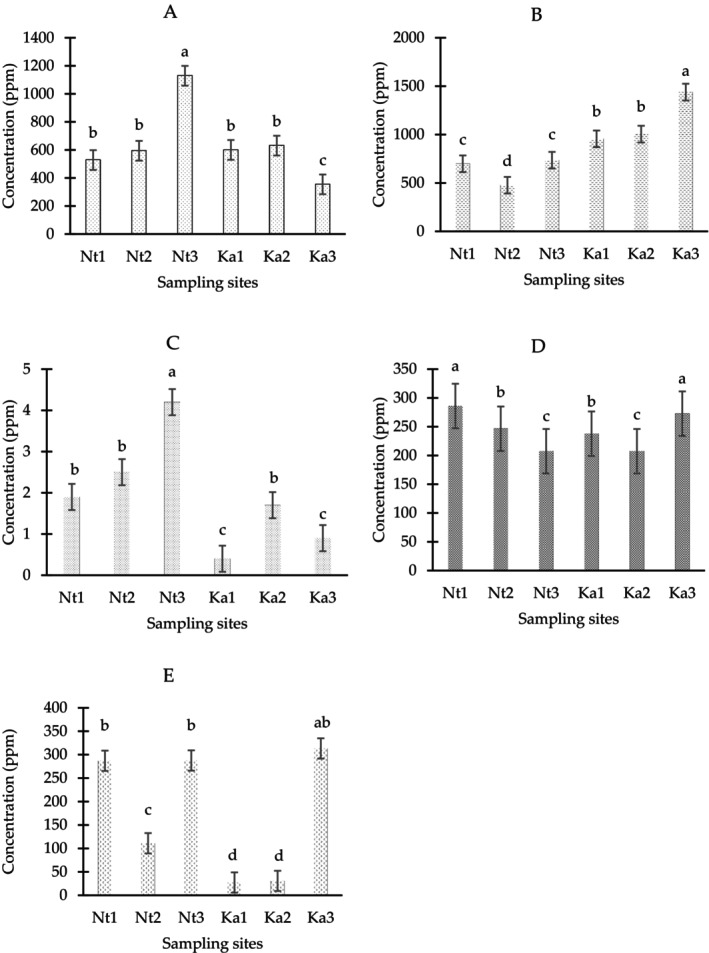
The total concentration (ppm) of esters (A), alcohols (B), ketones (C), organic acids (D), and aldehydes (E) in traditional fermented *Tonto* samples from Ntungamo and Kalungu districts. The bar graphs represent the mean of two separate fermentations from the same sampling site while the error bars represent the standard deviation between two replicate determinations. All analyses were done in duplicate. Bars with different letters across sampling sites indicate differences among *Tonto* samples determined by Tukey's HSD test at 95% confidence level.

The concentrations of volatile compounds in *Tonto* produced in Ntungamo and Kalungu districts are presented in Figure 2. The volatiles quantified included esters, alcohols, ketones, acids, and aldehydes. These are known key contributors to the aroma and flavor profile of several fermented alcoholic beverages (Lyumugabe et al. 2018; Mukisa et al. 2017). Sample Nt3 from Ntungamo had significantly (*p* < 0.05) higher concentrations of esters (1128.8 ± 184 mg/L) (due to higher levels of ethyl acetate, 982.0 ± 0.01 mg/L) and ketones (4.2 ± 0.3 mg/L). Samples Ka3 from Kalungu had significantly (*p* < 0.05) higher concentrations of alcohols (1439.1 ± 86.1 mg/L) (due to high levels of methanol, 950.5 ± 3.21 mg/L), organic acids (272.7 ± 38.7 mg/L) and aldehydes (313.3 ± 21.6 mg/L) and significantly (*p* < 0.05) low levels of esters (354.6 ± 70.5 mg/L) and ketones (0.9 ± 0.3 mg/L). The study findings indicated that alcohols (Ntungamo at 634.0 ± 158.7 mg/L, Kalungu at 1093.3 ± 249.9 mg/L) and esters (Ntungamo at 764.4 ± 431.7 mg/L, Kalungu at 520.8 ± 189.9 mg/L) were the predominant volatiles in both districts, consistent with the metabolic activity of yeast during alcoholic fermentation. These were followed by organic acids (Ntungamo at 234.8 ± 60.0 mg/L, Kalungu at 221.7 ± 23.0 mg/L), aldehydes (Ntungamo at 217.1 ± 96.9 mg/L, Kalungu at 124.1 ± 177.1 mg/L) and ketones (Ntungamo at 2.7 ± 1.2 mg/L, Kalungu at 0.8 ± 0.6 mg/L). However, the alcohols were high in concentration in *Tonto* from Kalungu district which could give it a more intense aroma whereas the relative abundance of esters in *Tonto* from Ntungamo imparted a fruitier and more pleasant flavor that is associated with higher acceptability. These differences in abundance of volatiles highlight the regional diversity in *Tonto* production practices, microbial communities, fermentation conditions or banana cultivars used in production of these beverages (Lyumugabe et al. 2018).

In general, volatile compounds showed relatively smaller error bars suggesting consistent variations in their production during spontaneous fermentation. The degree of overlap among error bars varied across volatile groups, reflecting differences in variability among samples. However, statistical significance was assessed using Tukey's HSD test rather than visual inspection of error bars. Therefore, significant differences (*p* < 0.05) are indicated by different letters above the bars and may occur even when error bars overlap partially.

A total of thirty‐eight (38) volatile flavor compounds were identified in traditionally fermented *Tonto* from Ntungamo and Kalungu districts (Tables 2, 3, 4). These included 16 esters, 13 alcohols, 3 ketones, 5 acids and 1 aldehyde. Thirty‐four of the thirty‐eight were common across all the samples. Among the identified compounds, esters were the most abundant in all samples with concentrations ranging from 0.02 ± 0.00 mg/L to 982.0 ± 0.01 mg/L. The highest concentrations for ethyl acetate (982.0 ± 0.01 mg/L) were observed in *Tonto* obtained from Ntungamo (Nt3) and 366.8 ± 2.00 mg/L in Kalungu (Ka2), followed by isobutyl acetate, phenethyl acetate, and ethyl caprylate. Alcohols were also prominent, with isoamyl alcohol present in the highest concentration (202.1 mg/L) in Ntungamo (Nt3) and methanol highest in Kalungu (950.5 mg/L) (Ka3). Methanol was detected at levels ranging from 285.4 ± 0.97 to 950.5 ± 3.21 mg/L in Kalungu and 0 to 43.9 ± 0.89 mg/L in Ntungamo. Other notable compounds identified included acids such as butyric, acetic and hexanoic acids as well as ketones like diacetyl and pentanedione.

**TABLE 2:** Concentrations of esters in traditional fermented *Tonto* from Ntungamo and Kalungu at 24 h of fermentation.

Compounds	Concentrations of flavor compounds (mg/L)						OT (mg/L)	Odor activity values		Odor description
	Nt1	Nt2	Nt3	Ka1	Ka2	Ka3		Ntungamo	Kalungu	
Ethyl acetate	64.0^d^ ±0.02	276.8^c^ ±0.97	982.0^a^ ±0.01	356.8^b^ ± 0.33	366.8^b^ ± 2.00	ND	7.5^[1]^	58.8 ± 64.1	48.3 ± 0.9	Pineapple^[5]^
Ethyl butyrate	19.8^a^ ± 0.12	13.0^b^ ±2.02	ND	ND	ND	ND	0.02^[3]^	818.1 ± 240.3	—	Apple^[5]^
Isoamyl acetate	8.2^a^ ± 0.05	4.5^b^ ± 0.14	6.6^ab^ ± 0.23	3.0^c^ ± 1.01	4.0b^c^ ± 0.21	7.8^a^ ± 0.11	0.00015^[2]^	42868.9 ± 12513.4	32711.1 ± 16767.3	Banana^[5]^
Ethyl hexanoate	0.6^a^ ± 0.08	0.3^b^ ± 1.32	0.4^ab^ ± 0.41	0.3^b^ ± 2.12	0.5^ab^ ±3.24	0.5^ab^ ± 0.12	0.44^[11]^	1.0 ± 0.3	1.0 ± 0.3	Pineapple, fruity, waxy, banana, green^[11]^
Isobutyl acetate	140.2^a^ ± 1.24	91.1^ab^ ± 0.41	54.8^d^ ± 0.98	43.1^e^ ± 0.10	94.1^b^ ± 9.12	79.2^c^ ± 1.89	0.025^[2]^	3815.4 ± 1714.1	2886.7 ± 1049.0	Fruity, slightly bitter^[6]^
Ethyl succinate	12.1^c^ ± 0.03	5.4^d^ ± 3.03	18.0^b^ ± 0.44	ND	ND	22.0^a^ ± 2.1	200^[1]^	0.1 ± 0.0	0.1 ± 0.0	Faint, pleasant^[6]^
Ethyl caprylate	43.3^b^ ± 0.09	25.6^c^ ± 0.09	50.5^a^ ± 3.01	28.0^c^ ± 2.14	45.5^ab^ ± 0.00	33.2^c^ ± 4.43	0.002^[8]^	19898.5 ± 6419.8	17788.0 ± 4496.6	Fruity, floral^[8]^
Ethyl caproate	12.9^f^ ± 0.03	18.2^c^ ± 0.39	24.7^b^ ± 2.11	33.3^a^ ± 0.21	14.0^e^ ± 0.31	15.2^d^ ± 0.00	0.005^[8]^	3719.1 ± 1176.3	4163.2 ± 2168.2	Fruity, anise^[8]^
Methyl acetate	45.8^a^ ± 0.07	32.3^c^ ± 0.50	21.9^d^ ± 0.88	20.1^e^ ± 0.03	37.2^b^ ± 0.32^cd^	19.4^f^ ± 0.09	2.3^[1]^	14.5 ± 5.2	11.1 ± 4.4	Pleasant, fruity, slightly bitter^[6]^
Butyl acetate	1.9a^b^ ± 0.14	2.8^ab^ ± 0.09	1.7^ab^ ± 3.33	3.0^a^ ± 12.01	ND	1.2^ab^ ± 0.11	0.066^[3]^	32.2 ± 9.0	32.2 ± 19.0	Fruit, pear &banana^[6]^
Isoamyl butyrate	17.2^c^ ± 0.21	25.7^b^ ± 0.31	20.4^c^ ± 0.41	33.4^a^ ± 4.91	29.8^ab^ ± 2.89	40.0^a^ ± 0.08	0.015^[2]^	1405.0 ± 285.2	2292.7 ± 344.8	Fruity, apricot, pineapple, banana, sweet^[6]^
Phenethyl acetate	61.1^a^ ± 0.33	59.2^a^ ± 4.1	52.8^b^ ± 0.21	44.7^c^ ± 3.32	60.2^a^ ± 7.12	58.9^a^ ± 0.01	0.25^[2]^	230.7 ± 17.5	218.4 ± 34.3	Sweet fragrance^[7]^
Butyl butyrate	4.0^b^ ± 2.10	ND	ND	ND	ND	8.4^a^ ± 0.34	0.4^[2]^	10.0 ± 0.0	21.0 ± 0.0	Fruit, banana^[7]^
Hexyl acetate	1.2^b^ ± 0.99	3.3^a^ ± 2.40	2.2^b^ ± 2.21	1.3^b^ ± 3.00	1.4^b^ ± 3.21	3.2^a^ ± 0.67	0.002^[3]^	1130.2 ± 527.8	998.0 ± 539.5	Pleasant, fruity^[5]^
Ethyl ocatnoate	ND	ND	ND	2.0^a^ ± 0.22	0.6^b^ ± 0.04^a^	ND	19.3^[14]^	—	0.1 ± 0.0	Fruity, wine, waxy, sweet, brandy, pear^[14]^
Ethyl valerate	0.1^a^ ± 1.44	0.1^a^ ± 0.82	0.02^b^ ± 0.12	0.02^b^ ± 0.00	0.1^a^ ± 1.80	0.01^b^ ± 0.99	0.0058^[2]^	12.1 ± 8.1	5.0 ± 3.9	Fruit, apple^[7]^

*Note:* NB: OT – odor threshold; ND – not detected; OAV − not calculated; Odor threshold was obtained from literature: ^[1]^Xiao et al. (2016); ^[2]^Van Gemert (2011); ^[3]^Niu, Wang, Xiao, Zhu, et al. (2019); ^[8]^Guth (1997); ^[9]^Murnane et al. (2013); ^[10]^Olaniran et al. (2017); ^[11]^Wang et al. (2021); ^[13]^Schneider (2023); ^[14]^Wang et al. (2024); Odor descriptions were obtained from literatures: ^[4]^Wei et al. (2020); ^[5]^Niu, Wang, Xiao, et al. (2019); ^[6]^Burdock (2016); ^[7]^The Good Scents Company (n.d.); ^[8]^Guth (1997); ^[9]^Murnane et al. (2013); ^[10]^Olaniran et al. (2017); ^[11]^Wang et al. (2021); ^[13]^Zviely (2009); ^[14]^Wang et al. (2024). OAV was calculated by dividing concentration by the odor threshold value of the compound. The data represents the mean of two separate fermentations from the same sampling site and the value after ± represents the standard deviation between two replicate determinations. All analyses were done in duplicate. Compounds with different letters within the same row indicate differences among *Tonto* samples determined by Tukey's HSD test at 95% confidence level.

**TABLE 3:** Concentrations of alcohols in traditional fermented *Tonto* from Ntungamo and Kalungu at 24 h of fermentation.

Compounds	Concentrations of flavor compounds (mg/L)						OT (mg/L)	Odor activity values		Odor description
	Nt1	Nt2	Nt3	Ka1	Ka2	Ka3		Ntungamo	Kalungu	
Propanol	ND	ND	112.2^a^ ± 0.81	35.1^c^ ± 0.12	35.9^c^ ± 3.12	49.7^b^ ± 0.32	314^[1]^	0.4 ± 0.0	0.1 ± 0.1	—
n‐Butanol	2.0^c^ ± 2.12	1.8^c^ ± 2.31	4.1^a^ ± 3.33	2.2^c^ ± 0.19	3.1^b^ ± 0.82	2.9^b^ ± 0.42	240^[3]^	0.02 ± 0.0	0.01 ± 0.0	Plastics, perfumes^[6]^
2,3Methylbutanol	101.6^a^ ± 0.01	62.4^c^ ± 0.30	0.7^d^ ± 1.01	100.6^a^ ± 0.32	103.5^a^ ± 2.00	73.5^b^ ± 4.32	0.5^[1]^	109.8 ± 101.8	185.1 ± 33.1	Fruity^[7]^
Isoamyl alcohol	152.2^b^ ± 0.03	100.4^c^ ± 1.18	202.1^a^ ± 2.03	98.2^c^ ± 0.31	143.6^b^ ± 4.00	112.0^c^ ± 2.31	30^[8]^	5.1 ± 1.7	3.9 ± 0.8	Cheese^[8]^
Phenethyl alcohol	101.2^a^ ± 0.04	50.0^c^ ± 1.10	87.9^b^ ± 3.10	54.2^c^ ± 0.21	26.2^d^ ± 2.01	81.0^b^ ± 3.12	0.56^[2]^	142.4 ± 47.5	96.3 ± 49.1	Rose, sweet^[6]^
Methanol	43.9^c^ ± 0.89	ND	ND	285.4^b^ ± 0.97	288.2^b^ ± 3.01	950.5^a^ ± 3.21	3.05^[9]^	14.4 ± 0.0	167.4 ± 125	Sour, sweet, alcohol^[9]^
Pentanol	3.3^b^ ± 1.12	3.1^b^ ± 2.00	5.1^a^ ± 2.21	3.0^b^ ± 0.00		4.2^a^ ± 2.1	0.01^[1]^	386.1 ± 109.5	360.2 ± 86.9	Floral, fruity^[6]^
Hexanol	ND	1.0^a^ ± 0.30	1.1^a^ ± 3.01	ND	ND	ND	0.25^[3]^	4.4 ± 0.0	—	Herbaceous, fragrant, sweet, green, fruity^[6]^
Ethanol	59.2^c^ ± 0.062	99.2^a^ ± 2.19	101.8^a^ ± 2.00	59.5^c^ ± 0.12	70.4^b^ ± 0.41	66.2^b^ ± 3.00	0.01^[2]^	8673.2 ± 2386.7	6538.5 ± 548	Strong alcoholic, ethereal medical^[14][4]^
1‐Octanol	200.4^a^ ± 0.44	45.4^e^ ± 5.15	120.2^d^ ± 2.01	153.4^c^ ± 3.00	184.9^b^ ± 3.11	14.4^f^ ± 1.99	0.12^[8]^	1016.8 ± 646.0	527.8 ± 651	Waxy, green, orange, rose, mushroom
2‐Methyl‐1‐propanol	15.3^b^ ± 0.77	3.0^d^ ± 3.23	10.0^c^ ± 3.23	17.2^b^ ± 2.12	20.0^a^ ± 0.31	10.0^c^ ± 0.43	6.51^[2]^	1.5 ± 1.0	2.4 ± 0.8	Penetrating, wine‐like, disagreeable^[6]^
1‐Heptanol	1.0^a^ ± 4.23	1.2^a^ ± 0.33	0.1^c^ ± 1.12	0.6^b^ ± 3.23	2.0^a^ ± 0.32	1.1^a^ ± 3.91	5.4^[14]^	0.1 ± 0.1	0.2 ± 0.2	Violet herbal, green, sweet, woody, peony^[14]^
3‐Hexan‐1‐ol	0.03^a^ ± 0.20	0.02^a^ ± 0.43	0.04^a^ ± 3.01	0.04^a^ ± 0.00	0.02^a^ ± 4.19	0.04^a^ ± 4.12	0.11^[2]^	0.3 ± 0.1	0.3 ± 0.1	Green leaf, violet leaf^[6]^

*Note:* NB: OT – odor threshold; ND – not detected; OAV − not calculated; Odor threshold was obtained from literature: ^[1]^Xiao et al. (2016); ^[2]^Van Gemert (2011); ^[3]^Niu, Wang, Xiao, Zhu, et al. (2019); ^[8]^Guth (1997); ^[9]^Murnane et al. (2013); ^[10]^Olaniran et al. (2017); ^[11]^Wang et al. (2021); ^[13]^Schneider (2023); ^[14]^Wang et al. (2024); Odor descriptions were obtained from literatures: ^[4]^Wei et al. (2020); ^[5]^Niu, Wang, Xiao, et al. (2019); ^[6]^Burdock (2016); ^[7]^The Good Scents Company (n.d.); ^[8]^Guth (1997); ^[9]^Murnane et al. (2013); ^[10]^Olaniran et al. (2017); ^[11]^Wang et al. (2021); ^[13]^Zviely (2009); ^[14]^Wang et al. (2024). OAV was calculated by dividing concentration by the odor threshold value of the compound. The data represents the mean of two separate fermentations from the same sampling site and the value after ± represents the standard deviation between two replicate determinations. All analyses were done in duplicate. Compounds with different letters within the same row indicate differences among *Tonto* samples determined by Tukey's HSD test at 95% confidence level.

**TABLE 4:** Concentrations of ketones, acids and aldehydes in traditional fermented *Tonto* from Ntungamo and Kalungu districts.

Compounds	Concentrations of flavor compounds (mg/L)						OT (mg/L)	Odor activity values		Odor description
	Nt1	Nt2	Nt3	Ka1	Ka2	Ka3		Ntungamo	Kalungu	
Ketones										
Diacetyl	0.5^a^ ± 1.99	1.1^a^ ± 0.14	2.8^a^ ± 4.00	0.3^a^ ± 0.39	1.3^a^ ± 10.10	0.6^a^ ± 2.09	0.2^[10]^	9.7 ± 8.1	5.1 ± 3.4	Butterscotch^[10]^
Pentanedione	0.01^a^ ± 0.66	0.01^a^ ± 1.00	0.03^a^ ± 2.12	0.01^a^ ± 6.05	0.1^a^ ± 2.19	0.02^a^ ± 4.91	1.5^[13]^	0.01 ± 0.01	0.04 ± 0.1	Honey‐like^[13]^
Acetone	1.3^a^ ± 1.11	1.1^a^ ± 0.10	1.2^a^ ± 0.98	ND	ND	ND	0.4^[9]^	2.9 ± 0.2	—	sweet, fruity, etherous^[9]^
Acids										
Isobutyric acid	10.0^a^ ± 2.02	2.5^e^ ± 0.31	7.7^c^ ± 0.32	7.9^b^ ± 0.10	4.4^e^ ± 2.21	6.6^d^ ± 3.91	6.6	1.0 ± 0.6	1.0 ± 0.3	Fresh floral, fruity, sweet, strawberry^[6]^
Acetic acid	259.1^a^ ± 0.31	190.2^c^ ± 0.88	163.3^d^ ± 3.12	201.2^b^ ± 0.30	184.0^c^ ± 2.91	208.1^b^ ± 8.88	200.0^[1]^	1.0 ± 0.3	1.0 ± 0.1	Pungent, vinegar^[6]^
Butyric acid	0.2^a^ ± 1.00	1.0^a^ ± 0.12	3.2^a^ ± 3.89	1.1^a^ ± 2.01	0.9^a^ ± 3.34	0.9^a^ ± 3.81	0.2^[3]^	8.7 ± 9.2	5.8 ± 0.7	Penetrating, sweet^[6]^
Hexanoic acid	1.0^a^ ± 2.31	0.9^a^ ± 0.20	0.2^a^ ± 3.12	0.2^a^ ± 3.12	1.1^a^ ± 2.00	0.02^a^ ± 7.07	0.4^[3]^	1.6 ± 1.0	1.0 ± 1.4	Characteristic pineapple^[6]^
Octanoic acid	29.7^a^ ± 0.18	28.1^b^ ± 0.32	7.2^e^ ± 0.15	10.1^c^ ± 0.34	8.9^ce^ ±11.12	29.7^a^ ± 0.10	0.5^[1]^	43.4 ± 25.0	32.4 ± 23.3	Pleasant, fruity, floral^[6]^
Aldehydes										
Acetaldehyde	271.0^c^ ± 0.04	105.2^d^ ± 0.40	275.1^b^ ± 0.26	21.4^e^ ± 4.00	22.4^e^ ± 3.09	328.6^a^ ± 5.05	0.5^[3]^	434.2 ± 194	248.3 ± 354	Yogurt, alcoholic^[4]^

*Note:* NB: OT – odor threshold; ND – not detected; OAV − not calculated; Odor threshold was obtained from literature: ^[1]^Xiao et al. (2016); ^[2]^Van Gemert (2011); ^[3]^Niu, Wang, Xiao, Zhu, et al. (2019); ^[8]^Guth (1997); ^[9]^Murnane et al. (2013); ^[10]^Olaniran et al. (2017); ^[11]^Wang et al. (2021); ^[13]^Schneider (2023); ^[14]^Wang et al. (2024); Odor descriptions were obtained from literatures: ^[4]^Wei et al. (2020); ^[5]^Niu, Wang, Xiao, et al. (2019); ^[6]^Burdock (2016); ^[7]^The Good Scents Company (n.d.); ^[8]^Guth (1997); ^[9]^Murnane et al. (2013); ^[10]^Olaniran et al. (2017); ^[11]^Wang et al. (2021); ^[13]^Zviely (2009); ^[14]^Wang et al. (2024). OAV was calculated by dividing concentration by the odor threshold value of the compound. The data represents the mean of two separate fermentations from the same sampling site and the value after ± represents the standard deviation between two replicate determinations. All analyses were done in duplicate. Compounds with different letters within the same row indicate differences among *Tonto* samples determined by Tukey's HSD test at 95% confidence level.

The flavor profile of *Tonto* revealed a complex mixture dominated by esters and alcohols. These compounds are well‐known contributors to the fruity and floral notes of fermented fruit beverages respectively (Lyumugabe et al. 2018). Isoamyl acetate, with the highest odor activity value (OAV) (32711.1 ± 16767.3 to 42868.9 ± 12513.4) emerged as the key aroma‐active compound imparting banana‐like notes, consistent with findings in other tropical fruit wines and beers (Chen et al. 2020; Lyumugabe et al. 2018). Odor Activity Value refers to the ratio of the concentration of a volatile compound in a sample to its sensory odor threshold concentration. OAVs are commonly used to assess the relative contribution of individual volatile compounds to the overall aroma profile, where compounds with OAV values greater than 1 are generally considered to contribute perceptibly to the aroma characteristics of the product (Lyumugabe et al. 2018). Esters such as ethyl butyrate, ethyl caprylate, and ethyl caproate further contributed to fruity and sweet flavors through esterification reactions between organic acids and alcohols during alcoholic fermentation (Saerens et al. 2010). Alcohols like 2,3‐methylbutanol (185.1 ± 33.1 to 109.8 ± 101.8), isoamyl alcohol (3.9 ± 0.8 to 5.1 ± 1.7) and phenethyl alcohol (96.3 ± 49.1 to 142.4 ± 47.5) also had high OAVs indicating that they have significant contributions to the aroma of *Tonto* in addition to being precursors for ester formation. High levels of isoamyl alcohol confer fruity, winey and fusel breath flavor whereas the phenyl ethanol has rose and sweet flavor (Chen et al. 2020; Englezos et al. 2018). Ethanol was the most significant alcohol present at high concentrations and possessing high odor activity values. In fermented products, ethanol is responsible for the characteristic flavor as well as the solubility and perception of other volatiles.

Methanol levels ranging from 43.9 to 950.5 mg/L were detected in some *Tonto* samples, with 50% of the samples exceeding the Uganda National Bureau of Standards specification for *Tonto* (US 2143:2019; limit 60 mg/L). This contamination was markedly more prevalent in Kalungu (285.4 ± 0.97 to 950.5 ± 3.21 mg/L) than in Ntungamo (0–43.9 ± 0.89 mg/L), indicating regional differences in processing practices. High methanol levels (563.8–953.1 mg/L) have also been reported in banana wine (Byarugaba‐Bazirake et al. 2013). Methanol contamination in traditional fermentations has been associated with poor hygiene, rudimentary equipment, mixed microbial cultures, contamination, and use of pectin‐rich substrates (Alvarenga et al. 2011; Ohimain 2016). Processors in this study reported using unripe or unpeeled bananas during juice extraction, which inadvertently increases methanol risk because pectin degradation (via Pectin Methyl Esterase activity) by microorganisms such as *Saccharomyces cerevisiae, Bacillus subtilis, Aspergillus niger* and *Erwinia carotovora* can produce methanol (Chaiyasut et al. 2013). This practice was reportedly more common in Kalungu, consistent with the higher methanol concentrations observed. Moreover, fruit‐derived precursors such as glycine may be metabolized by yeasts and contribute to methanol formation (Liang et al. 2015; Shen et al. 2024). Importantly, methanol poisoning may present after a delay (commonly 12–24 h) and can be further delayed when ethanol is co‐ingested, which can reduce early recognition and delay treatment seeking (Najari et al. 2020; Zavuga et al. 2023). This is relevant for traditional beverages like *Tonto* that are consumed socially and may be co‐consumed with other ethanol‐containing drinks. Therefore, the elevated methanol levels detected in Kalungu samples (up to 950.5 mg/L) indicate a potential hazard for consumers, and the risk could be amplified if *Tonto* is subsequently distilled to produce local spirits (e.g., waragi), where methanol may concentrate in early fractions due to poor distillation practices (Gensi et al. 1996; Ohimain 2016). On consumption, methanol is converted to formaldehyde and formic acid which are poisonous to the optic nerves and the nervous system potentially causing blindness and death (Liberski et al. 2022).

The elevated methanol levels observed here present an urgent public health concern since Uganda has experienced recurrent, documented methanol poisoning episodes linked to adulterated or unsafe alcoholic beverages. A Uganda Public Health Institute bulletin summarized prior fatal events, including 5 deaths in Kasese in 2005, 40 deaths in Kampala in 2007, and 7 deaths in Kasana, Kampala in 2009 attributable to methanol poisoning (Tusiime et al. 2017). In April 2010, a major episode in southwestern Uganda (Kabale District) was reported to have caused approximately 80 deaths after consumption of alcoholic beverages adulterated with methanol (Rwakahangi et al. 2025). An outbreak investigation in Wakiso District (June 2017) documented deaths following consumption of locally distilled alcohol adulterated with methanol, with clinical features consistent with methanol toxicity (Birungi et al. 2020). More recently in Arua City and Madi‐Okollo District (August 2022), an outbreak linked to contaminated “Gin X” recorded 48 cases and 18 deaths (38% Case Fatality Ratio) and reported an average symptom onset of approximately 13 h after last ingestion (Zavuga et al. 2023). Collectively, these outbreak records show that methanol contamination is a persistent safety issue in Uganda and reinforce the need for practical control measures to minimize methanol formation in traditional banana‐based beverages.

Acids detected included octanoic, butyric, acetic, hexanoic and isobutyric acids whereas the ketones identified included acetone, diacetyl and pentanedione. Although detected in lower concentrations, hexanoic and butyric acid contributed to perceptible flavor notes due to their low odor thresholds (0.42 and 0.17 mg/L respectively). These short and medium chain fatty acids produced via yeast lipid metabolism can enhance the aroma complexity of *Tonto*. However, in excessive amounts, these compounds can be associated with off‐flavors or spoilage. Diacetyl and acetaldehyde were also identified in *Tonto*. These compounds are known to impart milk flavor in fermented products which may be undesirable in case of *Tonto*. Some compounds had low concentrations across all samples but their OAVs were high due to their low odor threshold values. These included isoamyl acetate, ethyl caprylate, isobutyl acetate, ethyl caproate, isoamyl butyrate, phenethyl acetate, hexyl acetate, 2,3methylbutanol, phenethyl alcohol, ethanol and 1‐octanol. Similarly, these compounds were reported at threshold and suprathreshold levels in banana wine and *Urwagwa* (Chen et al. 2020; Lyumugabe et al. 2018).

In comparison, *Urwagwa* and *Tonto* share ten major compounds with OAVs > 1. These are: ethyl butyrate, ethyl acetate, isoamyl acetate, ethyl caprylate, ethyl caproate, phenethyl acetate, phenethyl alcohol, and 1‐octanol (Lyumugabe et al. 2018). In their findings, the fruity notes of *Urwagwa* were associated with ethyl esters such as ethyl caprylate, ethyl caprate, ethyl caproate, ethyl butyrate, and ethyl acetate whereas the floral and banana notes were associated with acetate esters like isoamyl acetate, phenethyl acetate, ethyl caprylate, phenethyl alcohol, and 1‐octanol. The overall aroma of *Urwagwa* was dominated by fruity notes due to high amounts of ethyl caprylate, ethyl caprate, and ethyl caproate (Lyumugabe et al. 2018).

Although the overall volatile profile was similar across both regions, Ntungamo *Tonto* generally exhibited higher concentrations and OAVs for key esters and alcohols. This suggests potential differences in fermentation conditions, microbial communities, or banana juice composition between the two regions. Such variation can influence consumer perception and may present opportunities for regionally branded sensory profiles of *Tonto*.

### 3.3 Sensory Properties

**FIGURE 3:**
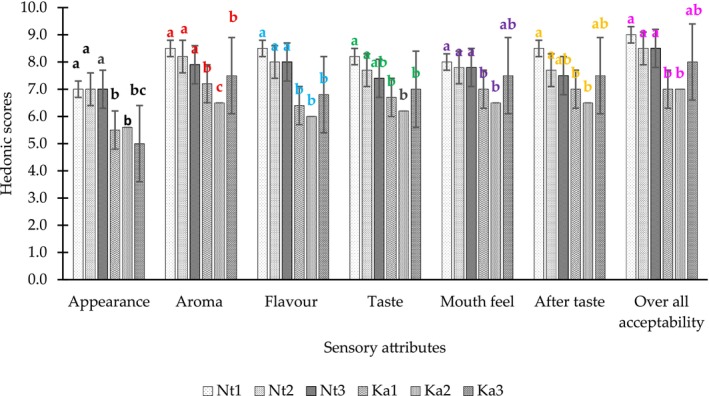
Mean hedonic scores of traditional fermented *Tonto* samples from Ntungamo and Kalungu districts. The bar graphs represent the mean scores of two separate fermentations from the same sampling site, while the error bars represent the standard deviation between two replicate determinations. All analyses were done in duplicate. Bars with different letters within the same group indicate differences among *Tonto* samples determined by Tukey's HSD test at a 95% confidence level.

The mean hedonic scores of *Tonto* samples at 24 h fermentation are presented in Figure 3. The results revealed significant variations among the *Tonto* samples with respect to appearance, aroma, flavor, taste, mouthfeel, aftertaste, and overall acceptability. Generally, samples from Ntungamo received higher (*p* < 0.05) sensory scores than those from Kalungu. In particular, sample Nt1 exhibited the highest scores across most sensory attributes and overall acceptability, suggesting superior sensory quality and consumer preference. This may be attributed to its well‐balanced flavor profile, characterized by desirable volatile compounds such as isoamyl acetate, ethyl acetate, phenethyl acetate, phenethyl alcohol, ethyl caprylate, and ethyl caproate, which contribute fruity, floral, and characteristic banana‐like aromas and flavors. Higher concentrations of these compounds are known to enhance sensory quality and consumer preference. In particular, alcohols and esters contribute floral and fruity notes; however, excessively high concentrations may negatively affect flavor quality and overall sensory perception (Andorrà et al. 2010; Moreira et al. 2008).

The beer banana varieties traditionally used in Ntungamo and Kalungu contributed significantly to differences in the sensory quality and fermentation characteristics of *Tonto*. East African Highland Beer Bananas commonly used in Ntungamo are characterized by higher tannin, latex, and phenolic contents, resulting in *Tonto* with a stronger bitter‐astringent taste, fuller body, darker color, and more complex flavor profile (Karamura and Karamura 1994; Lyumugabe et al. 2012). These phenolic compounds and tannins also influence yeast activity, fermentation dynamics, and sensory complexity during alcoholic fermentation (Corona et al. 2021; Rinaldi et al. 2016). *Tonto* from Kalungu was generally sweeter, less bitter, lighter in body, and more acidic due to the use of banana varieties such as Kayinja, which are characterized by lower tannin and latex contents, higher fermentable sugar levels, and softer pulp texture (Kyamuhangire et al. 2002). The higher sugar availability and lower tannin content may promote the growth of acid‐producing microorganisms during spontaneous fermentation, leading to increased organic acid production and sour flavor development (Lyumugabe et al. 2012; De Vuyst and Leroy 2020). These varietal differences also influenced microbial succession, fermentation efficiency, alcohol formation, and the physicochemical properties of the final product.

Most sensory attributes exhibited relatively small error bars, indicating good agreement among panelists. The few larger error bars observed for aroma, flavor, and overall acceptability suggest greater differences in individual perception of these attributes, which is expected because these characteristics are influenced by personal sensory sensitivity and preference. Despite this variability, significant differences among samples were confirmed by Tukey's HSD test, as indicated by the superscript letters.

**FIGURE 4:**
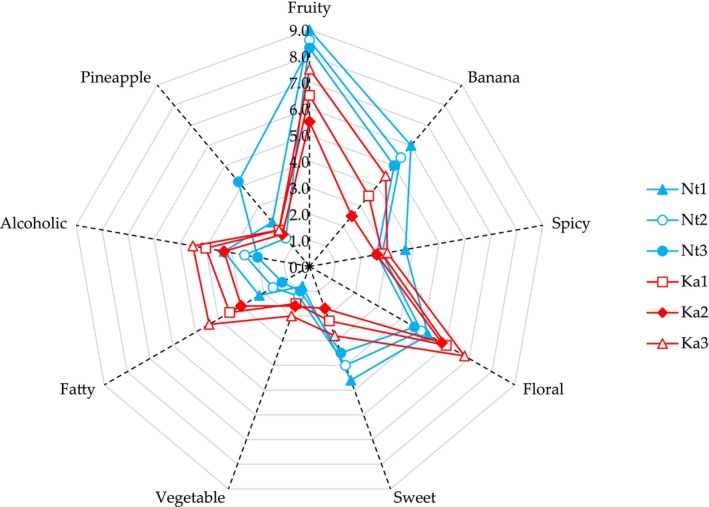
Sensory profile of traditional fermented *Tonto* from Ntungamo and Kalungu districts. The results are mean scores of two separate fermentations from the same sampling site.

The sensory attributes of *Tonto* samples from Ntungamo and Kalungu districts at 24 h of fermentation were evaluated by a trained panel of 10 assessors using a 9‐point quantitative line scale. The radar chart in Figure 4 shows the mean scores for sensory attributes of *Tonto*. The study findings indicated that the *Tonto* obtained from Ntungamo consistently scored higher values (*p* < 0.05) across most sensory attributes. The high scores for fruity and banana attributes in sample Nt1 (Figure 4) agree with the elevated concentrations and odor activity values of esters such as isoamyl acetate, ethyl hexanoate, butyl acetate, isoamyl butyrate, butyl butyrate, ethyl caprylate, ethyl caproate, and ethyl acetate that are strongly associated with banana and fruity aromas (Figure 2A, Table 2). These attributes are most preferred and translated into a higher overall acceptability observed in samples from Ntungamo than in those from Kalungu district (Figure 3).

In contrast, the Kalungu *Tonto* samples received higher ratings only in a few attributes, such as floral, fatty, alcoholic, and vegetable, which were rated similarly or slightly better than Ntungamo. These sensory characteristics may correspond to different fermentation by‐products or regional differences in raw materials or microbial flora. Attributes such as spicy and pineapple received relatively low scores in both samples, suggesting these notes were either minimal or not favored by the panel. The high scores in fatty, vegetable, and alcoholic notes observed in Kalungu samples were due to high concentrations of alcohols. These were responsible for the low acceptability scores obtained in samples from this region (Figure 3).

### 3.4 Relationship Between Sensory Properties, Physico‐Chemical Composition and Aroma Compounds of *Tonto* From Ntungamo and Kalungu

#### 3.4.1 Principal Component Analysis

**FIGURE 5:**
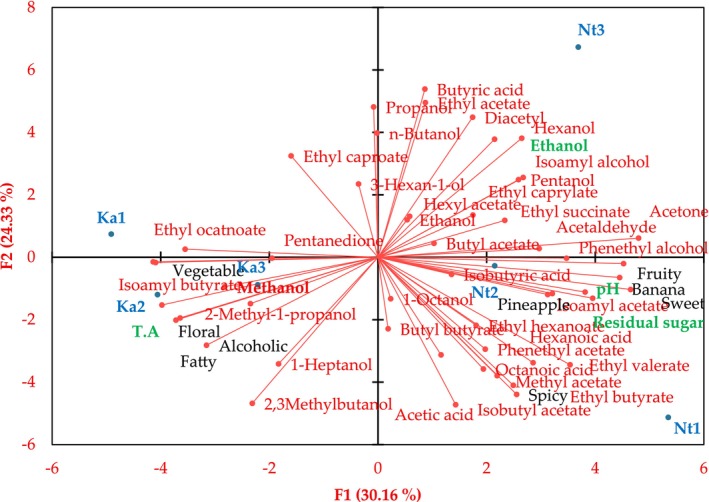
Biplot of aroma compounds, sensory attributes and physicochemical properties of traditional fermented *Tonto* from Ntungamo (Nt1–Nt3) and Kalungu (Ka1–Ka3) districts.

Principal Component Analysis (PCA) was performed to explore relationships between the sensory attributes, physicochemical parameters and aroma‐active compounds in *Tonto* samples from Ntungamo and Kalungu districts. As shown in Figure 5, the first two principal components (F1 and F2) explained 30.16% and 24.33% of the total variance, respectively, accounting for a cumulative 54.49% of the variability in the dataset. Although the cumulative explained variance (54.49%) was moderate, this level is considered acceptable for exploratory multivariate analysis of complex food systems involving physicochemical, sensory, and volatile compound datasets, where variability is often distributed across multiple interacting biological and processing factors rather than concentrated in only a few principal components (Granato et al. 2018). In food fermentation systems, moderate explained variance in PCA is common because aroma formation, physicochemical properties, and sensory characteristics are influenced simultaneously by raw material composition, microbial activity, fermentation conditions, and processing practices (Granato et al. 2018). Therefore, the PCA biplot was primarily used to identify clustering patterns and relationships among variables rather than explain all variability in the dataset. The remaining unexplained variance may reflect additional contributing factors including microbial diversity, fermentation dynamics, site‐specific processing practices, and raw material variation.

*Tonto* samples obtained from Ntungamo district were located in the right upper (Nt3) and lower (Nt1 and Nt2) quadrants whereas those from Kalungu were located in the left upper (Ka1) and lower (Ka2 and Ka3) quadrants (Figure 5).

Principal Component 1 (F1), which accounted for the largest proportion of variance (30.16%) separated *Tonto* samples based on the intensity of fruity, banana, sweet, and pineapple attributes. These descriptors clustered on the right side of the biplot, closely associated with key esters such as ethyl butyrate, isoamyl acetate, ethyl caprylate, and ethyl hexanoate, as well as alcohols like ethanol and phenethyl alcohol. This pattern is consistent with the Ntungamo sample's profile, which scored higher in these sensory attributes and had higher Odor Activity Values (OAVs) for these volatiles.

F2 (24.33%) distinguished variables such as ethyl caproate and ethyl octanoate, which are more associated with strong flavors. This axis also captured variation in acetic acid and methyl acetate, linking them to possible sharp or solvent‐like notes.

In contrast, attributes such as alcoholic, vegetable, floral, and fatty were positioned negatively along both F1 and F2. They also showed closer association with compounds like methanol, 2‐methyl‐1‐propanol, 1‐heptanol, 2,3‐methylbutanol, and isoamyl butyrate. These components were more aligned with the Kalungu samples, which received lower acceptability scores and lower concentrations of desirable fruity esters. Physicochemical parameters like pH and residual sugar aligned more closely with esters and fruity sensory descriptors, suggesting a link between sweetness perception and ester formation. On the other hand, total acidity correlated with vegetable and alcoholic notes, implying that increased acidity may enhance sour or vegetal characteristics. The PCA analysis reinforces the conclusion that fruity and banana‐like aroma compounds, particularly esters, are strongly associated with positive sensory perception and overall acceptability of *Tonto*. These compounds are closely aligned with *Tonto* samples obtained from Ntungamo district, explaining their higher hedonic scores. In contrast, compounds associated with vegetal, fatty, or solvent‐like aroma notes, such as those observed in the Kalungu samples, may contribute to lower sensory appeal.

#### 3.4.2 Hierarchical Cluster Analysis (HCA)

**FIGURE 6:**
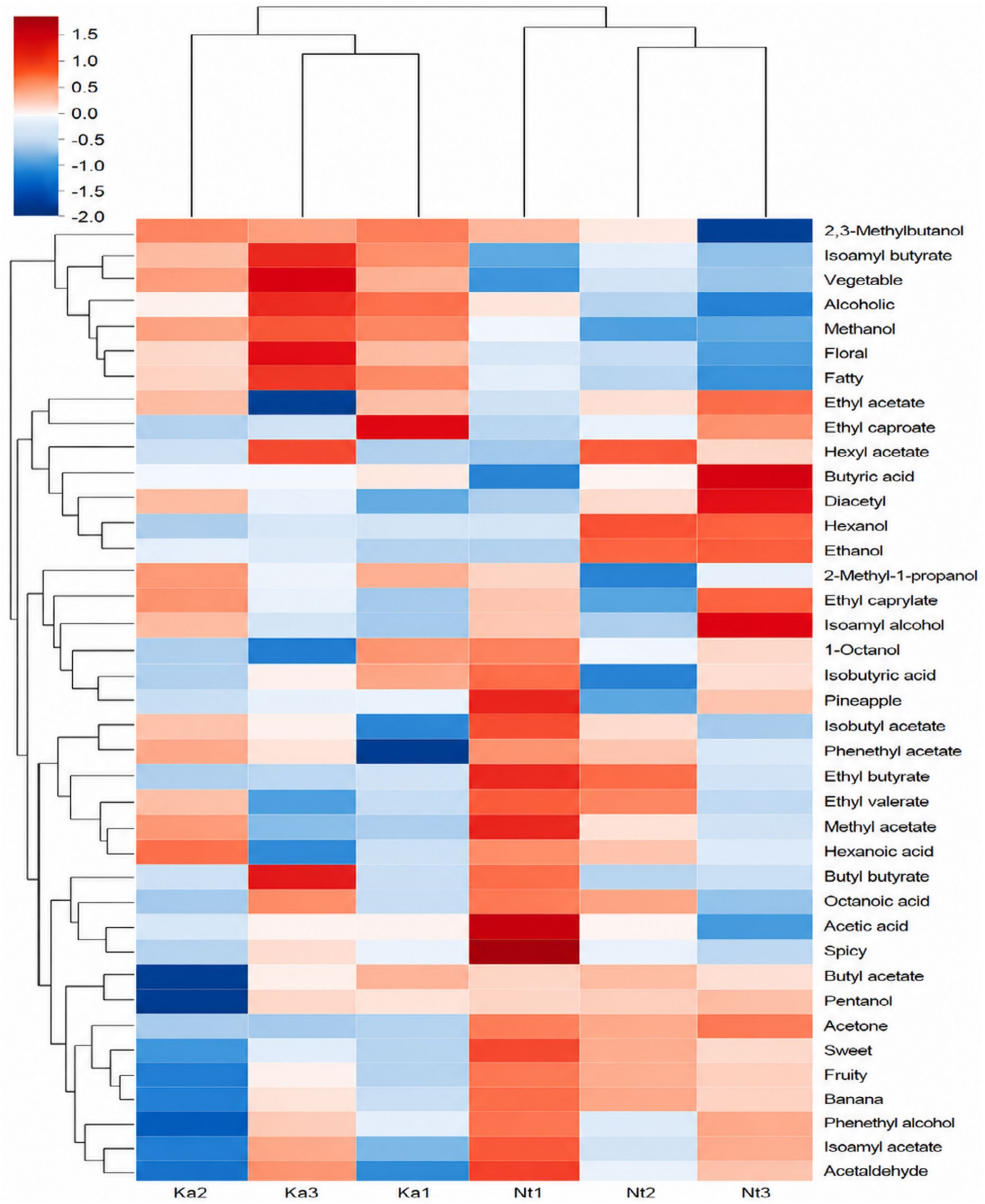
Hierarchical clustering and heat map visualization of volatile compounds and sensory attributes of traditional fermented *Tonto* samples from Ntungamo and Kalungu districts.

Hierarchical cluster analysis (HCA) was performed on volatile compounds with odor activity values (OAVs) > 1 together with sensory aroma descriptors, and the results were visualized using a heat map (Figure 6). Prior to clustering, data were log‐transformed and standardized (*z*‐score) to minimize the influence of scale differences among variables. In the heat map, red and blue colors represent relatively higher and lower expression levels, respectively, compared to the mean value of each compound or sensory attribute across all samples.

The column‐wise clustering separated the *Tonto* samples from the six sampling sites into two distinct groups according to geographical origin. Samples from Ntungamo (Nt1, Nt2 and Nt3) clustered together and were characterized by relatively higher expression of volatile flavor compounds and positive aroma attributes, whereas samples from Kalungu (Ka1, Ka2 and Ka3) formed a separate cluster and generally exhibited lower relative intensities of these compounds. This pattern corroborates the analytical results of the present study, which showed higher concentrations of aroma‐active compounds in Ntungamo samples compared to those from Kalungu.

Row‐wise clustering revealed chemically and sensorially meaningful groupings of volatile compounds and aroma notes. One cluster was dominated by alcohols, including methanol, 2‐methyl‐1‐propanol, and 2,3‐methylbutanol, together with a limited number of esters such as butyl butyrate and isoamyl butyrate. This cluster also included vegetable, alcoholic, floral, and fatty aroma notes, which generally received lower sensory scores. These attributes are commonly associated with elevated concentrations of alcohols and have been reported to negatively influence sensory quality when present at high levels (Andorrà et al. 2010; Moreira et al. 2008). Compounds and aroma notes in this cluster were more pronounced in Kalungu samples, which were also less preferred by consumers.

The second major cluster was dominated by ethyl and acetate esters, fatty acids, ketones, aldehydes, and selected alcohols. This group co‐clustered with fruity, banana, pineapple, sweet, and spicy aroma notes, which are typically associated with enhanced flavor intensity and overall acceptability. These compounds were relatively more abundant in Ntungamo samples, which also received higher sensory scores and greater acceptability. Esters and fatty acids are well known contributors to desirable aroma characteristics in fermented products (Benito et al. 2015; Houtman et al. 1980).

Overall, the hierarchical clustering of volatile compounds together with sensory attributes demonstrates a strong association between chemical composition and sensory perception. The clear separation of samples by geographical origin further highlights the influence of site‐specific fermentation conditions on aroma development and acceptability of *Tonto*.

## 4 Conclusions

Regional variations in fermentation practices, banana varieties, and climatic conditions in Ntungamo and Kalungu significantly influenced the physicochemical properties, fermentation dynamics, and sensory quality of *Tonto*. *Tonto* from Ntungamo, produced mainly from traditional East African Highland beer bananas, exhibited a more bitter‐astringent taste, fuller body, darker color, and relatively high alcohol content due to the higher tannin, latex, and phenolic composition of the raw materials. In contrast, *Tonto* from Kalungu, commonly produced from *Kayinja* and other juice‐rich cultivars, was generally sweeter, lighter in body, and more acidic because of higher fermentable sugar availability and lower tannin content. These varietal differences influenced microbial activity, favoring ethanologenic fermentation in Ntungamo and acid‐producing microorganisms in Kalungu, ultimately affecting flavor development, fermentation efficiency, and overall product quality.

## Author Contributions

**Abubakar Sadik Mustafa:** validation, software, writing – review and editing, supervision, resources, formal analysis. **Ambrose Atwine:** conceptualization, investigation, writing – original draft, methodology, validation, visualization, software, formal analysis, data curation. **Stellah Byakika:** methodology, software, formal analysis, visualization, writing – review and editing, supervision. **Albert Nuwagira:** methodology, formal analysis, software, writing – review and editing, validation. **Ivan Muzira Mukisa:** resources, conceptualization, investigation, writing – review and editing, supervision. **Abel Atukwase:** conceptualization, investigation, writing – review and editing, supervision, data curation. **Charles M. B. K. Muyanja:** investigation, writing – review and editing, supervision, resources, project administration.

## Ethics Statement

The authors have nothing to report.

## Consent

Written informed consent was obtained from all study participants.

## Data Availability

The data that support the findings of this study are available from the corresponding author upon reasonable request.
